# Genes involved in complex adaptive processes tend to have highly conserved upstream regions in mammalian genomes

**DOI:** 10.1186/1471-2164-6-168

**Published:** 2005-11-27

**Authors:** Soohyun Lee, Isaac Kohane, Simon Kasif

**Affiliations:** 1 Bioinformatics Program, Boston University, Boston, MA 02215, USA; 2 Department of Biomedical Engineering, Boston University, Boston, MA 02215, USA; 3 Center for Advanced Genomic Technology,. Boston University, Boston, MA 02215, USA; 4 Children's Hospital Informatics Program at Harvard-MIT Health Sciences and Technology, Boston, MA 02215, USA

## Abstract

**Background:**

Recent advances in genome sequencing suggest a remarkable conservation in gene content of mammalian organisms. The similarity in gene repertoire present in different organisms has increased interest in studying regulatory mechanisms of gene expression aimed at elucidating the differences in phenotypes. In particular, a proximal promoter region contains a large number of regulatory elements that control the expression of its downstream gene. Although many studies have focused on identification of these elements, a broader picture on the complexity of transcriptional regulation of different biological processes has not been addressed in mammals. The regulatory complexity may strongly correlate with gene function, as different evolutionary forces must act on the regulatory systems under different biological conditions. We investigate this hypothesis by comparing the conservation of promoters upstream of genes classified in different functional categories.

**Results:**

By conducting a rank correlation analysis between functional annotation and upstream sequence alignment scores obtained by human-mouse and human-dog comparison, we found a significantly greater conservation of the upstream sequence of genes involved in development, cell communication, neural functions and signaling processes than those involved in more basic processes shared with unicellular organisms such as metabolism and ribosomal function. This observation persists after controlling for G+C content. Considering conservation as a functional signature, we hypothesize a higher density of *cis*-regulatory elements upstream of genes participating in complex and adaptive processes.

**Conclusion:**

We identified a class of functions that are associated with either high or low promoter conservation in mammals. We detected a significant tendency that points to complex and adaptive processes were associated with higher promoter conservation, despite the fact that they have emerged relatively recently during evolution. We described and contrasted several hypotheses that provide a deeper insight into how transcriptional complexity might have been emerged during evolution.

## Background

Transcription regulation is among the most sophisticated of regulatory processes, involving a complex combinatorial selection of *cis*- and *trans*-acting signals [1]. Proximal upstream regions of a gene in particular contain many *cis*-regulatory elements that regulate the expression of the gene by binding to various transcription factors. Many of the *cis*-regulatory motifs have been successfully identified by phylogenetic footprinting, which makes use of cross-species sequence conservation as a functional signature [2-6]. Based on this rationalization, we aimed to test if the complexity of transcriptional regulation depends on gene function, by looking at the sequence conservation at the proximal upstream region. This is the first whole genome study providing statistical evidence of significant conservation in upstream regions of human genes involved in certain biological processes and functions.

There have been studies on how gene function is related to degree of conservation and evolutionary rate in the protein-coding region. For example, Clark et al. [7] detected functional categories that showed accelerated evolution of the protein-coding region in human compared to chimp, based on site-specific dN/dS ratios. The functional categories include 'olfaction', 'signal transduction', 'cell adhesion', 'transport', 'developmental processes', 'ion channel' and 'extracellular matrix'. In [8], Dorus et al. reported that the protein coding regions of the genes linked to nervous system development show positive selection in the primate lineage compared to rodents, by using housekeeping genes as a control. Bustamante et al. [9] compared within-species polymorphism and between-species divergence to detect positively and negatively selected human genes. Functional categories with an excess of positively selected genes included 'sensory perception', 'defence/immunity protein' and in particular, 'transcription factor'. Some discrepancy exists among studies, since [9] detected 'ectoderm development', 'extracellular matrix' and 'voltage-gated potassium channel' as categories with an excess of negatively selected genes.

Little is known about how the proximal upstream regions evolve in those genes that are detected to be positively selected, even though it is an interesting question whether the noncoding regulatory regions will show a similar pattern as the coding regions. Interestingly, we detected a significantly higher upstream conservation in these adaptive genes, particularly those involved in development, cell communication, signal transduction, transcription factor and neurophysiological functions. One possibility is that there is opposite purifying selection on the upstream regulatory region and the protein-coding region. The other possibility is that a relatively high regulatory complexity exists in these genes and that their dense *cis*-regulatory elements provide higher promoter conservation. We speculate that the latter explanation is biologically more intuitive. According to [10], a complex network can rapidly achieve the ability to buffer mutations. One interesting connection we can make is that the genes in a complex network tend to evolve more rapidly in the sequence level because their mutations can be buffered more easily.

Thus, in this paper, we provide a possible insight on the combinatorial complexity of the transcriptional regulation and its evolutionary meanings of the most complex and adaptive processes such as development and cell communication.

## Results

We identified functional categories that are enriched towards high proximal promoter conservation by computing a Spearman's rank correlation on upstream sequence alignment scores and Gene Ontology (GO) [11] terms.

**Figure 1:**
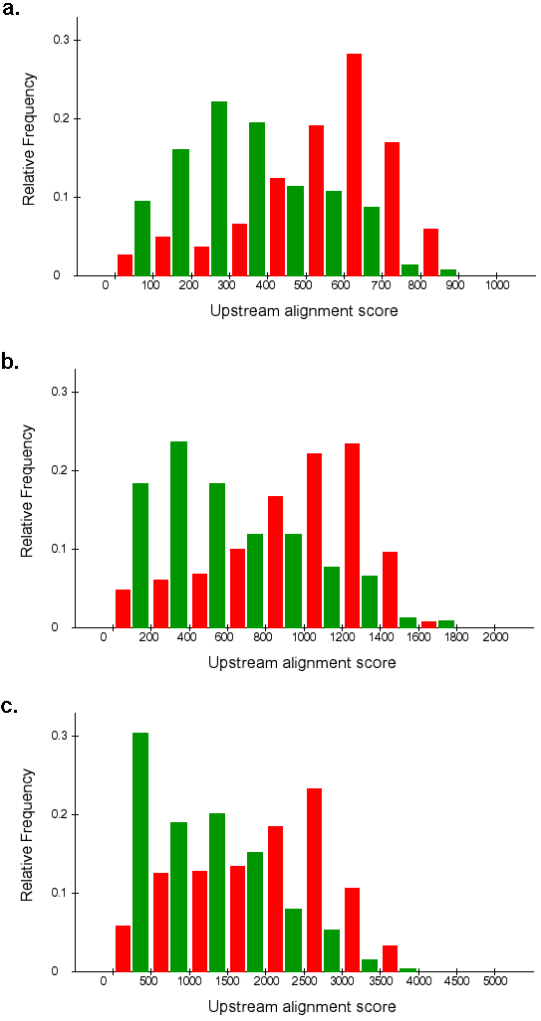
**Distribution of alignment scores of selected terms**. Histograms of alignment scores of genes annotated with selected terms that were significantly correlated with high or low alignment scores. a: 1 kb upstream, red: neurogenesis (+), green: ribosome (-), b. 2 kb upstream, red: neurogenesis (+), green: ribonucleoprotein complex (-), c. 5 kb upstream, red: neurogenesis (+), green: ribonucleoprotein complex (-), where (+) and (-) represents positively and negatively correlated with high upstream alignment scores, respectively.

The terms 'development', 'morphogenesis' and 'organogenesis' were found at the top of the list, followed by 'cell communication', 'signal transduction', 'transcription factor activity' and 'neurophysiological process'. Interestingly, more routine processes such as 'biosynthesis' and 'ribosome' turned out to be correlated with low upstream alignment scores (Table 1, 2, Additional file 1: Table 1). When we compared the alignment scores of negatively correlated terms vs. the most positively correlated terms, we found a 50% increase in the 1 kb, 2 kb and 5 kb alignment scores from former to the latter. The differences in alignment scores are clearly noticeable in the histograms of alignment scores of selected terms provided in Figure 1.

**Table 1:** Selected GO terms significantly enriched toward high 2 kb upstream alignment scores. P-values are Bonferroni-corrected. b,m,c represents GO hierarchy (b:biological process, m:molecular function, and c:cellular component). mean: mean alignment score. The mean alignment score for all the genes analyzed is 411.01.

GO accession		GO term definition	p-value	mean	# genes
GO:0007275	b	development	2.69E-48	860.98	1432
GO:0009653	b	morphogenesis	2.82E-47	898.06	949
GO:0009887	b	organogenesis	1.50E-42	911.47	765
GO:0048513	b	organ development	1.50E-42	911.47	765
GO:0007154	b	cell communication	8.30E-38	799.48	2473
GO:0007165	b	signal transduction	4.61E-24	791.28	1969
GO:0007399	b	neurogenesis	7.04E-22	945.20	322
GO:0003700	m	transcription factor activity	1.63E-20	892.63	673
GO:0050877	b	neurophysiological process	4.29E-09	836.34	435
GO:0019226	b	transmission of nerve impulse	7.07E-06	888.41	197
GO:0007268	b	synaptic transmission	1.07E-05	889.12	192
GO:0016055	b	Wnt receptor signaling pathway	4.19E-04	989.45	78

**Table 2:** GO terms significantly enriched toward low 2 kb upstream alignment scores. P-values are Bonferroni-corrected. b,m,c represents GO hierarchy (b:biological process, m:molecular function, and c:cellular component). mean: mean alignment score. The mean alignment score for all the genes analyzed is 411.01.

GO accession		GO term description	p-value	mean	# genes
GO:0005840	c	ribosome	2.74E-05	501.18	154
GO:0030529	c	ribonucleoprotein complex	2.69E-04	551.19	269
GO:0008270	m	zinc ion binding	3.03E-04	639.02	1089
GO:0003723	m	RNA binding	9.62E-04	579.31	410
GO:0003735	m	structural constituent of ribosome	1.21E-03	531.85	174
GO:0046914	m	transition metal ion binding	6.70E-03	647.13	1229
GO:0006952	b	defense response	1.86E-02	612.35	694
GO:0044249	b	cellular biosynthesis	2.00E-02	622.59	805
GO:0005739	c	mitochondrion	2.71E-02	610.17	529
GO:0003824	m	catalytic activity	3.43E-02	661.43	3900
GO:0009058	b	biosynthesis	3.92E-02	625.43	840

In order to confirm that the phenomenon we observed in human/mouse proximal promoter conservation is also observed with respect to other mammalian genomes and is not specific to human and mouse, we conducted a similar analysis based on human and dog genomes. The same key terms were found to be significantly correlated. (Additional file 2: Table 2). Also, we obtained very similar results by employing a different score function (S_L_) that penalizes sparsely distributed matches (details provided in the Method section). Thus, the significance is not an artifact of the global alignment scores that can be affected by random matches that are not functional motifs.

**Figure 2:**
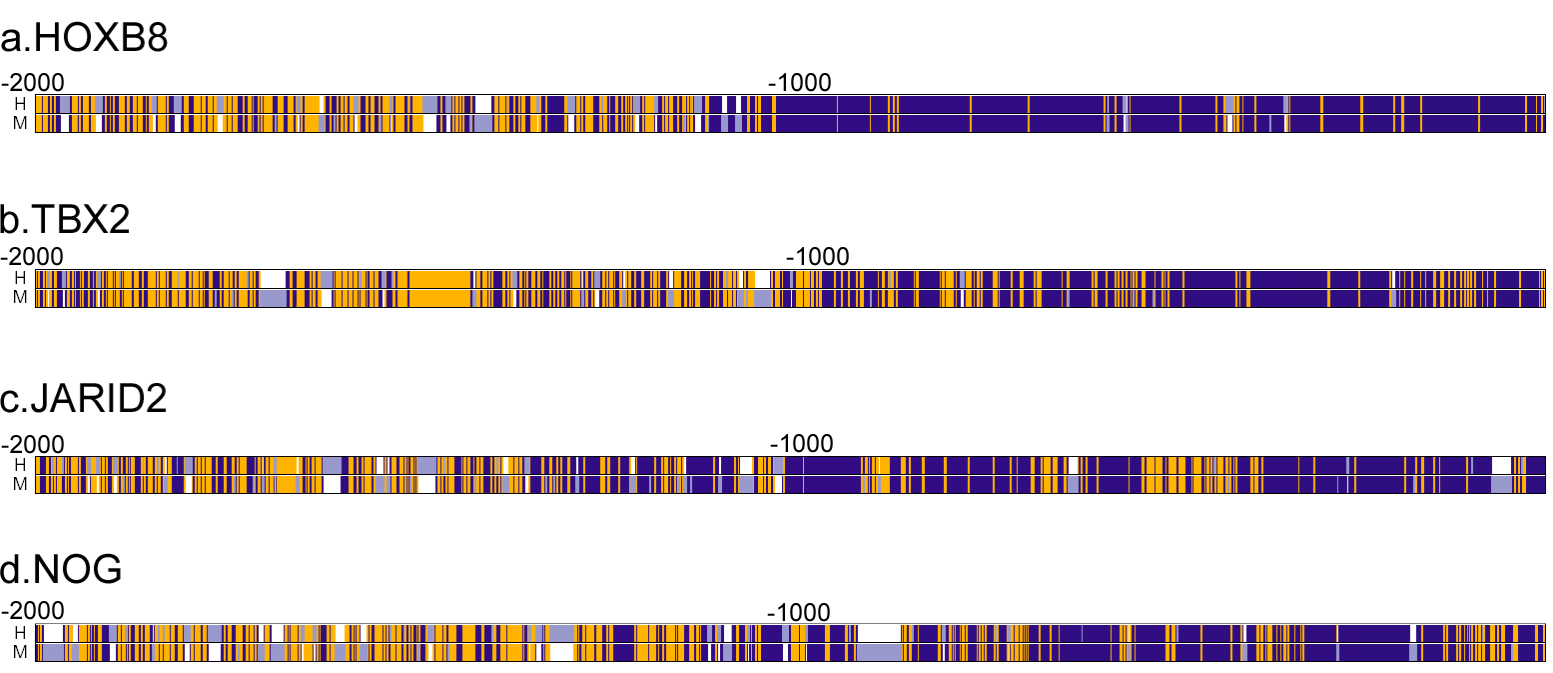
**Visualization of human-mouse alignments of 2 kb upstream of selected development genes**. The rightmost points are transcription start sites. **a. ***HOXB8 *(development, transcription factor), **b. ***TBX2 *(development, transcription factor), **c. ***JARID2 *(central nervous system development), **d. ***NOG *(neurogenesis, skeletal development). Blue: match, yellow: mismatch, white: gap.

Among developmental genes, we noticed that 36 *Hox *or *Hox *homologue genes (listed in Additional file 3: Table 3) had very high alignment scores (mean = 699.28, 1359.2 and 3235.6, two-sample KS-test p-value (two-tailed) = 9.19 × 10^-17^, 1.68 × 10^-17 ^and 2.65 × 10^-17 ^for 1 kb, 2 kb and 5 kb upstream, respectively). Differential expression of *Hox *genes confers positional identities in developing cells by forming sharp boundaries along the antero-posterior axis, and the strong conservation of the promoter region is very well expected. Genes involved in embryonic patterning such as *SHH *and *PTCH *also showed high promoter conservation (2 kb scores 1262 and 1522, respectively). Figure 2 visualizes the upstream alignments of selected development genes with top alignment scores.

Many transcription factors play important roles in development. There are a significantly large number of developmental genes among transcription factors, based on the GO annotation (215 out of 674, χ^2 ^test p-value = 7.82 × 10^-85^), indicating a larger number of both *trans*-elements as well as *cis*-elements in the developmental gene network. Then, is it transcription factors that contribute most to the enrichment of development? In order to test this, we performed rank correlation tests using only developmental genes. From the analysis based on 1 kb scores, only the term 'transcription factor activity' was enriched at the top (mean = 597.37, p = 4.43 × 10^-3^). However, in similar analyses on 2 kb and 5 kb, no significant term was identified, indicating that genes other than transcription factors must also contribute to the significantly higher upstream conservation of development. Even for 1 kb upstream regions, the significant enrichment of 'development' is not exclusively due to transcription factor genes, because a rank correlation test without the 674 transcription factor genes also gave strong significance for development. (p = 5.24 × 10^-28^) (similar for 2 kb and 5 kb regions).

Cell communication is known to play an important role in development [12]. However, cell communication and signal transduction remained significant after rank tests without developmental genes, indicating the significant correlation was not because of the developmental genes that also participate in these processes.

It has been reported that ultraconserved non-coding regions (UCRs) are located around key developmental regulator genes [13,14], which may suggest a complex developmental regulatory network spanning large chromosomal regions. However, most of the UCRs are far from genes, and few proximal promoter regions overlap with UCRs, suggesting that the complexity of regulation suggested by proximal promoter conservation is independent of UCR-mediated regulation.

The alignment score can be affected by G+C content (G+C%). Mutational biases that depend on base composition may affect the baseline conservation and it is easier to get a match in an alignment with high or low G+C%. Indeed, G+C% was positively correlated with alignment scores and even with the terms 'development', 'cell communication' and 'regulation of transcription' (data not shown). It can also affect the relationship between function and promoter conservation, because promoter type depends on G+C%. Also, a high G+C% is indicative of a higher neutral substitution rate, due to a higher rate of mutation at methylated cytosines in CpG sites and some other factors [15,16]

We performed a partial rank correlation [17] between upstream alignment score and GO term label, controlling for G+C%, to eliminate the effect of G+C% in the correlation test. There was no significantly enriched term for 1 kb upstream after the partial correlation, but for 2 kb and 5 kb, the key terms detected remained, indicating G+C% generally had little effect on our results (Additional file 4: Table 4).

A high upstream G+C% may also reflect a potential CpG island, which plays a role in transcriptional repression in a variety of types of cells and tissues. To compare regulation by CpG island methylation and regulation via other *cis*-regulatory elements, we performed a rank correlation test between CpG dinucleotide frequency in 1 kb, 2 kb and 5 kb upstream regions of human genes and GO term label. The result was somewhat different from the alignment score/functional label analysis, in that terms related to metabolism, cell cycle and transcription were found to be positively correlated and terms related to response to external signal, immune response, membrane and extracellular matrix were negatively correlated with high CpG frequency (Additional file 5: Table 5).

## Discussion

A commonly invoked heuristic for discovery of functional sites is locating regions of high similarity across multiple species. Thus, a relatively high proportion of such conserved regions may indicate an increased number of functional *cis*-elements. This in turn may suggest a more complex combinatorial circuitry in the transcriptional regulatory network, since higher density of functional *cis*-elements will allow more combinations of *trans*-acting signals as well.

In this context, our results indicate the existence of a relatively high complexity in the transcriptional regulation of development, cell communication, signal transduction, transcriptional regulation and neurophysiological function as compared to those of ribosomes and metabolism. This explanation is supported by prior gene-specific studies. For example, the sea urchin gene *Endo16 *has been found to have a dense distribution of *cis*-elements. *Endo16 *is expressed in the endoderm and may play a role in cell adhesion. Its upstream sequence has been well characterized to have complex *cis*-regulatory modules [18] and the proximal promoter region was shown to be highly conserved during evolution [19].

However, a caveat of the analysis is the interpretation of alignment data. A match in an alignment is not an accurate indicator of either purifying selection or functionality of that site, since non-functional sites under neutral evolution can remain unchanged by vertical inheritance or multiple/reverse mutation. However, the effect of neutral mutation can be ignored in this analysis. Neutral mutations that have occurred between human and mouse lineage can be considered to be saturated, because of the sufficiently high evolutionary distance between these species [20]. The G+C%-partialling analysis described in the Result section also suggests that the difference in neutral substitution rate has little effect on our results.

Stronger purifying selection on the promoter *cis*-elements of complex genes is another alternative explanation. It is not easy to tell if a conserved region is from a single highly important functional site or from overlaps of less important functional sites. An increased percent identity in a genomic region may indicate more important functional elements (stronger purifying selection) rather than a larger number of functional elements residing in that region.

In the classic Waddington's canalization theory, purifying (e.g. stabilizing) selection is a driving force for developmental genes to attain robustness against genetic and environmental changes [21]. In this context, developmental genes may undergo stronger purifying selection than others (Plotkin JB, personal communication). However, a recent view provided by [10] denies the necessity of stabilizing selective pressure to attain robustness for a system that can be represented as an interacting network. Developmental [10] and nervous system [22] can be represented as a network and thus the genes involved in these systems can intrinsically achieve the buffering ability. The finding that protein-coding regions of these genes undergo rapid evolution in humans [7-9] may indicate that the complexity of their network can buffer the mutations in the sequence level more easily. Our regulatory complexity hypothesis is consistent with the network implementation of canalization in developmental genes. Multiple regulatory sites interacting with increased number of trans-factors can achieve increased network connectivity. Although we find this explanation more biologically intuitive than the hypothesis that the higher purifying selection on the genes that has positive evolution in the protein-coding region, purifying selection and regulatory complexity might not be entirely disjoint and both factors can contribute to the conservation of promoter sequences as well as to their buffering capacity.

Another alternative hypothesis is that the transcriptional control of development genes is modulated by a family of transcription factors that require an increased specificity or a longer binding site. Indeed, many developmental regulators act as dimers that can take up larger areas on DNA. However, dimerization can also be considered a part of gene regulation (e.g. different heterodimeric combinations, dominant negative-type repression, etc.) and therefore would support the complex regulation hypothesis.

Recently, a similar study [23] was performed in yeast (that lacks developmental, neural or complex cell communication mechanisms) and it was reported that steroid, alcohol and carbohydrate metabolisms tend to be associated with higher promoter conservation in yeast. However, after a more careful examination of the entire GO term list provided in the Supplementary Material of [23], we found that the terms 'transcription factor activity', 'signal transduction', 'cell communication' and 'cellular morphogenesis' were associated with higher promoter conservation, whereas 'structural constituents of ribosome', 'DNA recombination' and 'RNA processing' had insignificant but negative enrichment, showing a consistent pattern with our results in mammals.

It has been suggested that a large fraction of genetic components in the evolution of development involves changes in transcriptional regulation [24]. One possible explanation of the link between complex regulation and adaptation is that adaptive changes in complex processes tend to occur in an incremental fashion, by slowly adding to its regulatory complexity, so that other important parts of the process remain functionally intact.

## Conclusion

We identified a class of functions that are associated with either high or low promoter conservation in mammals. We detected a significant tendency that complex and adaptive processes were associated with higher promoter conservation, despite the fact that they have emerged relatively recently during evolution. We described and contrasted several hypotheses that provide a deeper insight into how transcriptional complexity might have been emerged during evolution.

## Methods

### Promoter alignment and GO term labelling

To measure proximal promoter conservation, upstream alignment scores of human genes were computed by counting the number of matches in the alignment between the sequence 1 kb, 2 kb and 5 kb upstream of the transcription start site and its syntenic mouse sequence. In the cases in which a human gene had no recorded mouse counterpart, the score was set to 0. The alignments were obtained directly from the UCSC genome browser [25] (version Jun-2003). Genes with multiple promoter assignments were not included in the analysis, to avoid bias due to alternative promoters or incorrect annotation of transcription start sites. Consequently, 14449, 14434, 14412 genes were assigned alignment scores for 1 kb, 2 kb and 5 kb upstream, respectively.

GO term labeling was done as follows: for each of the 17594 non-obsolete GO terms, each gene was labeled 1 if it is annotated with the term itself or any of its descendants and 0 otherwise. Thus, each term was represented by a binary vector whose size is the number of the genes. We used the latest version of GO annotation downloaded on March 2005. The alignment scores and GO annotations of all the genes analyzed can be found in Additional file 6: Table 6.

### Rank correlation test

A Spearman rank correlation test [26] was performed on each GO term vector and the upstream alignment score vector, in order to test whether each GO term is associated with high or low proximal upstream conservation. Two-tailed p-values were calculated using Student's t-distribution. All the p-values provided in this paper and additional files are Bonferroni-corrected and the significance level used was α = 0.05.

### Human vs. dog

We used multi-species (human, chimp, dog, mouse, rat, chicken, zebrafish and fugu) upstream alignments downloaded from the UCSC genome browser (version May 2004) and extracted the human and dog alignment. The scoring scheme and correlation method were the same as in the human-mouse analysis described above.

### Modification of the alignment scores

Throughout the study described in the paper, we have used a simple global alignment score that might fail to capture a variety of features that represent patterns of conservation such as locally clustered functional regions. Thus, we tried a modified alignment score S_L _that penalizes sparsely distributed matches compared to locally enriched 'match blocks'.

S_L _= (# of matches) – (# of non-match blocks) + 1,

where a non-match block means consecutive runs of non-matches (mismatches or gaps) flanked by matches.

The last 1 is added to shift the score to be nonnegative. This score becomes 0 either when there is no match or all the matches are of length 1. The longer the blocks of matches are, the larger S_L _is.

## Authors' contributions

SL carried out the analysis and drafted the initial manuscript. SL, IK and SK interpreted the data and wrote portions of the final manuscript. SK coordinated the research. All authors read and approved the final manuscript.

## Supplementary Material

Additional File 1 Table 1 GO terms significantly correlated with upstream alignment scores. The table lists all the GO terms significantly correlated with 1 kb, 2 kb and 5 kb upstream alignment scores in the human-mouse analysis.
Click here for file

Additional File 2 Table 2 GO terms significantly correlated with upstream alignment scores in the analysis using dog. The table lists all the GO terms significantly correlated with 1 kb, 2 kb and 5 kb upstream alignment scores in the human-dog analysis. Click here for file

Additional File 3 Table 3 List of Hox and Hox homolog genes. List of Hox and Hox homolog genes used in the analysis, with their 1 kb, 2 kb and 5 kb upstream alignment scores. Click here for file

Additional File 4 Table 4 GO terms significantly correlated with upstream alignment scores after partialling out G+C%. The table lists all the GO terms significantly correlated with 1 kb, 2 kb and 5 kb upstream alignment scores in the human-mouse analysis after controlling G+C% using partial rank correlation. Click here for file

Additional File 5 Table 5 GO terms significantly correlated with CpG dinucleotide frequency in the human upstream sequence. The table lists all the GO terms significantly correlated with CpG dinucleotide frequency in the 1 kb, 2 kb and 5 kb human upstream sequences. Genes are sorted by 1 kb upstream alignment score. Click here for file

Additional File 6 Table 6 Human genes with their upstream alignment scores and Gene Ontology annotations. The 1 kb, 2 kb and 5 kb upstream alignment scores based on human-mouse comparison and Gene Ontology annotations of human genes. Click here for file
